# Brain age prediction using combined deep convolutional neural network and multi-layer perceptron algorithms

**DOI:** 10.1038/s41598-023-49514-2

**Published:** 2023-12-16

**Authors:** Yoonji Joo, Eun Namgung, Hyeonseok Jeong, Ilhyang Kang, Jinsol Kim, Sohyun Oh, In Kyoon Lyoo, Sujung Yoon, Jaeuk Hwang

**Affiliations:** 1 Ewha Brain Institute, Ewha Womans University, Seoul, South Korea; 2 Asan Institute for Life Sciences, Asan Medical Center, Seoul, South Korea; 3 Department of Radiology, Incheon St. Mary’s Hospital, College of Medicine, The Catholic University of Korea, Seoul, South Korea; 4 Department of Brain and Cognitive Sciences, Ewha Womans University, Seoul, South Korea; 5 Graduate School of Pharmaceutical Sciences, Ewha Womans University, Seoul, South Korea; 6 Department of Psychiatry, Soonchunhyang University College of Medicine, Seoul, South Korea

**Keywords:** Cognitive ageing, Computational neuroscience, Neural ageing

## Abstract

The clinical applications of brain age prediction have expanded, particularly in anticipating the onset and prognosis of various neurodegenerative diseases. In the current study, we proposed a deep learning algorithm that leverages brain structural imaging data and enhances prediction accuracy by integrating biological sex information. Our model for brain age prediction, built on deep neural networks, employed a dataset of 3004 healthy subjects aged 18 and above. The T1-weighted images were minimally preprocessed and analyzed using the convolutional neural network (CNN) algorithm. The categorical sex information was then incorporated using the multi-layer perceptron (MLP) algorithm. We trained and validated both a CNN-only algorithm (utilizing only brain structural imaging data), and a combined CNN-MLP algorithm (using both structural brain imaging data and sex information) for age prediction. By integrating sex information with T1-weighted imaging data, our proposed CNN-MLP algorithm outperformed not only the CNN-only algorithm but also established algorithms, such as brainageR, in prediction accuracy. Notably, this hybrid CNN-MLP algorithm effectively distinguished between mild cognitive impairment and Alzheimer’s disease groups by identifying variances in brain age gaps between them, highlighting the algorithm’s potential for clinical application. Overall, these results underscore the enhanced precision of the CNN-MLP algorithm in brain age prediction, achieved through the integration of sex information.

## Introduction

Chronological aging is intricately linked to several neurodegenerative conditions, including cognitive impairments and dementia^1^. During the natural aging process, the human brain experiences gray matter (GM) atrophy along with cortical thinning^2,3^. Despite these commonalities, the aging process of the human brain exhibits significant biological complexity and demonstrates marked inter-individual differences in both its rate and pattern^4,5^. Furthermore, underlying pathologies may hasten brain aging, and the individual brain aging may be differently influenced by both genetic and environmental factors for each person^4–6^.

Machine learning techniques that utilize brain magnetic resonance imaging (MRI) data can take these variations into account, thereby enhancing the accuracy in predicting an individual's brain age. The estimated brain age at an individual level serves as a personalized indicator for potential brain dysfunction^7–9^. Additionally, the difference between the predicted brain age and the chronological age, referred to as the "brain age gap", has emerged as a promising biomarker for detecting inter-individual differences in brain aging^7–9^. As the positive or negative brain age gap can respectively indicate accelerated or healthy brain aging, individual quantification of this gap may aid in both risk screening and the diagnostic process for neurodegenerative diseases^7,9^.

As structural MRI of the brain can detect aging-related neuroanatomical changes, such as global GM atrophy, it has been established that the chronological ages of healthy individuals can be accurately estimated using data from structural brain MRI^3,5,10^. These aging-related changes in brain structures differ according to gender^11^. Given that individual brain structures reflect both male and female characteristics in complex and dynamic patterns, the quantification of these patterns using machine learning should take into consideration these sex-specific brain characteristics^12^. Despite the understanding of the sex-specific trajectory of brain aging, there is a paucity of brain age prediction models that incorporate sex information as a relevant feature^13^. Instead, most of the current algorithms for brain age prediction utilizing structural brain MRI data tend to consider sex information only in the subsequent statistical correction process^14^.

In order to accurately predict brain age based on structural brain MRI data, the selection of suitable types of input data and algorithms for the optimal learning of normal brain aging patterns is essential^9,14^. From an algorithmic perspective, high-dimensional regression models employing deep neural networks have increasingly been utilized for brain age estimation^7,8,15,16^. Additionally, recent deep learning investigations have employed hybrid algorithms, incorporating numerical and/or categorical data to enhance prediction accuracy^17–19^. Specifically, the convolutional neural network (CNN), widely utilized in brain age prediction, has been recognized as optimal for interpreting highly complex brain structures^20,21^, and the supplementary use of the multi-layer perceptron (MLP) may offer advantages in terms of computing efficiency, depending on the types of input data^17,22^.

The current study is designed to introduce a novel algorithm that integrates both CNN and MLP algorithms for the prediction of brain age using mixed inputs, including minimally preprocessed T1-weighted images and biological sex information. We hypothesized that this combined CNN-MLP approach may demonstrate superior performance over the CNN-only algorithm, which relies solely on T1-weighted images for predicting brain age. The model's performance was evaluated in the internal validation set (n = 301), and external validation set (n = 645). Additionally, the performance of the combined CNN-MLP algorithm will be juxtaposed with that of the brainageR and pyment algorithms^15,23^, the latter two being the most widely used and extensively validated algorithms based on structural brain MRI data^24,25^.

## Results

### Performance of the brain age prediction algorithms in the training and test sets

**Figure 1:**
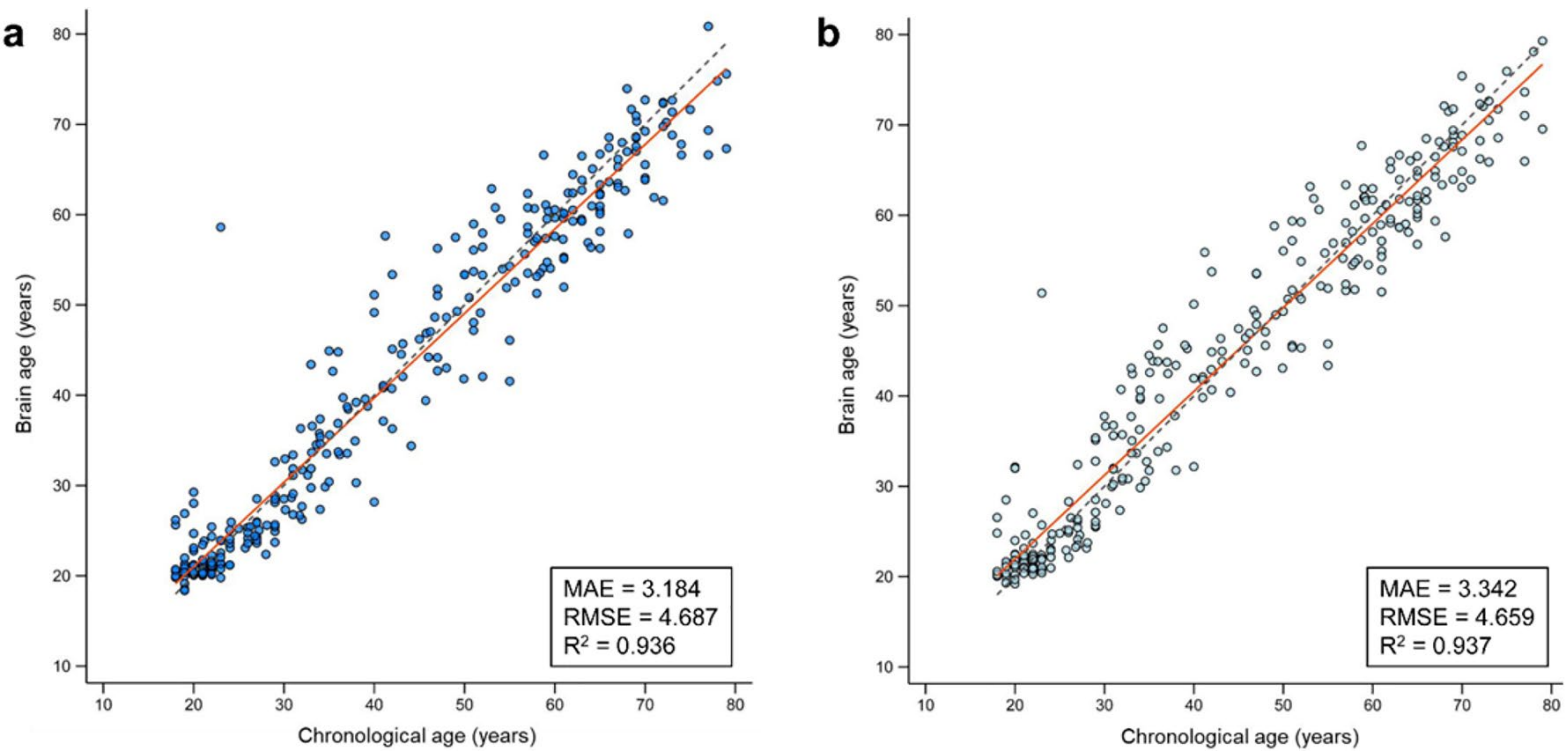
Comparison of the performance between the brain age prediction algorithms in internal validation set. Scatter plots show the predicted brain age versus chronological age in the internal validation set (test set) using the combined CNN-MLP algorithm (**a**, blue dots) and the CNN-only algorithm (**b**, gray dots). In all plots, the red line indicates a linear regression line and the dashed gray line indicates a y = x line (45-degree line). Abbreviations: CNN, convolutional neural network; MAE, mean absolute error; MLP, multi-layer perceptron; RMSE, root mean squared error; R^2^, coefficient of determination.

The predictive accuracy of both the combined CNN-MLP algorithm and the CNN-only algorithm, tested on the training set (n = 2703) and the test set (n = 301), is detailed in Table 1 and Fig. 1.

**Table 1:** Predictive accuracy of models employing the combined CNN-MLP algorithm and CNN-only algorithm.

	Algorithm	Input	Performance metrics		
			MAE (years)	RMSE (years)	R^2^
10-fold cross validation (Training set, n = 2,703)	Combined CNN-MLP	Minimally preprocessed whole brain T1-weighted image + sex information	3.494 ± 0.228	4.689 ± 0.570	0.933 ± 0.012
	CNN-only	Minimally preprocessed whole brain T1-weighted image	3.563 ± 0.193	4.839 ± 0.299	0.932 ± 0.009
Internal validation (Test set, n = 301)	Combined CNN-MLP	Minimally preprocessed whole brain T1-weighted image + sex information	3.184	4.687	0.936
	CNN-only	Minimally preprocessed whole brain T1-weighted image	3.342	4.659	0.937
External validation (CamCAN set, n = 645)	Combined CNN-MLP	Minimally preprocessed whole brain T1-weighted image + sex information	4.910	6.148	0.891
	CNN-only	Minimally preprocessed whole brain T1-weighted image	5.064	6.295	0.885

The results were obtained following the application of hyperparameter tuning, utilizing Adam as the chosen optimizer. Performance metrics from 10-fold cross-validation are presented as mean ± standard deviation.

*Adam* adaptive moment estimation, *CamCAN* Cambridge Centre for Ageing and Neuroscience, *CNN* convolutional neural network, *MAE* mean absolute error, *MLP* multi-layer perceptron, *RMSE* root mean squared error, *R*^*2*^ coefficient of determination.

In the training set, a 10-fold cross-validation of the proposed algorithm yielded the following outcomes: The combined CNN-MLP algorithm attained a mean MAE of 3.494 years, a mean RMSE of 4.689 years, and a mean R^2^ of 0.933. Conversely, the CNN-only algorithm reached a mean MAE of 3.563 years, a mean RMSE of 4.839 years, and a mean R^2^ of 0.932.

For the test set, the internal validation performance metrics were as follows: the combined CNN-MLP algorithm recorded an MAE of 3.184 years, an RMSE of 4.687 years, and an R^2^ of 0.936 (Fig. 1a), while the CNN-only algorithm achieved an MAE of 3.342 years, an RMSE of 4.659 years, and an R^2^ of 0.937 (Fig. 1b).

These results suggest that the combined CNN-MLP method, utilizing both minimally preprocessed T1-weighted images and sex information, outperformed the CNN-only algorithm that used only the minimally preprocessed T1-weighted image.

For further analysis, we assessed the efficiency of a more streamlined model, integrating a linear fully-connected layer at the end of the CNN model, capable of handling sex information. Within the training set, this model’s 10-fold cross-validation produced a mean MAE of 3.674 years, a mean RMSE of 5.042 years, and a mean R^2^ of 0.926. The test set performance metrics for the CNN integrated with a linear fully-connected layer were as follows: MAE of 3.592 years, RMSE of 4.989 years, and R^2^ of 0.927.

### Performance of the brain age prediction algorithms using the external validation set

**Figure 2:**
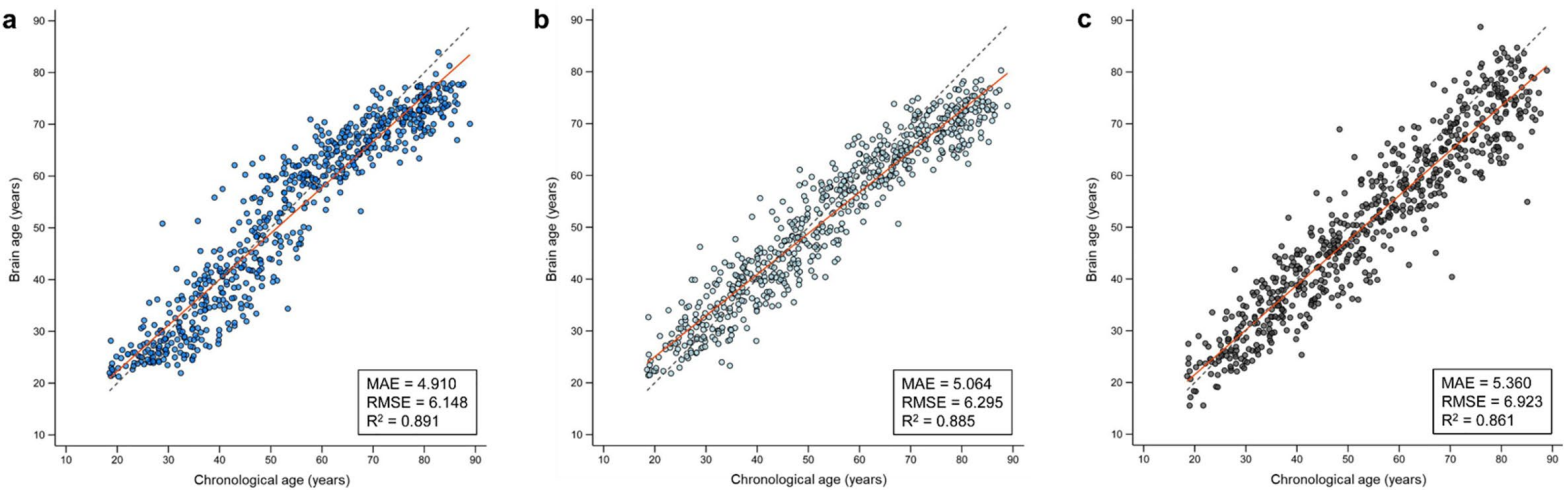
Comparison of the performance between the brain age prediction algorithms in external validation set. Scatter plots show the predicted brain age versus chronological age in the external validation set (CamCAN dataset) using the combined CNN-MLP algorithm (**a**, blue dots), CNN-only algorithm (**b**, light blue dots), and brainageR algorithm (**c**, black dots). In both plots, the red line indicates a linear regression line and the dashed gray line indicates a y = x line (45-degree line). Abbreviations: CamCAN, Cambridge Centre for Ageing and Neuroscience; CNN, convolutional neural network; MAE, mean absolute error; MLP, multi-layer perceptron; RMSE, root mean squared error; R^2^, coefficient of determination.

The predictive accuracy of the combined CNN-MLP algorithms in the external validation set is detailed in Table 2 and Fig. 2.

**Table 2:** Comparative predictive accuracy of our CNN-MLP and brainageR algorithms.

	Algorithm	Input	Performance metrics		
			MAE (years)	RMSE (years)	R^2^
Proposed algorithm	Combined CNN-MLP	Minimally preprocessed whole brain T1-weighted image + sex information	4.910	6.148	0.891
		Segmented GM & WM + sex information	5.276	6.452	0.879
brainageR ^15^	GPR	Segmented GM & WM	5.360	6.923	0.861

The performance of each algorithm was evaluated using the external validation dataset from the CamCAN set (n = 645).

*CamCAN* Cambridge Centre for Ageing and Neuroscience, *CNN* convolutional neural network, *GM* gray matter, *GPR* Gaussian process regression, *MAE* mean absolute error, *MLP* multi-layer perceptron, *RMSE* root mean squared error, *R*^*2*^ coefficient of determination, *WM* white matter.

In the external validation set (n = 645), which was sourced from the Cambridge Centre for Aging and Neuroscience (CamCAN) database (available at http://www.mrc-cbu.cam.ac.uk/datasets/camcan/), the combined CNN-MLP algorithm achieved an MAE of 4.910 years, an RMSE of 6.148 years, and an R^2^ of 0.891 (Fig. 2a); the CNN-only algorithm achieved an MAE of 5.064 years, an RMSE of 6.295 years, and an R^2^ of 0.885 (Fig. 2b). These findings consistently demonstrate that the combined CNN-MLP algorithm enhanced the predictive accuracy of brain age in the independent dataset.

We further compared the predictive accuracy of our combined CNN-MLP algorithm with the well-validated brainageR algorithm^15^ using an external validation set (n = 645) (Table 2, Fig. 2). The performance metrics for the brainageR algorithm included an MAE of 5.360 years, an RMSE of 6.923 years, and an R^2^ of 0.861 (Fig. 2c). Concurrently, retraining our combined CNN-MLP algorithm with the same inputs as the brainageR model, specifically the normalized gray matter and white matter images, resulted in an MAE of 5.276 years, an RMSE of 6.452 years, and an R^2^ of 0.879 (Table 2). These outcomes collectively suggest that our CNN-MLP model may offer superior predictive accuracy compared to the brainageR algorithm.

In addition, we compared the CNN-MLP algorithm with the pyment algorithm^23^, using a newly acquired dataset of healthy subjects (n = 200) from the Alzheimer's Disease Neuroimaging Initiative 1 (ADNI1, available at https://adni.loni.usc.edu/about/adni1/) and Open Access Series of Imaging Studies 1 (OASIS-1, available at http://www.oasis-brains.org/) databases. This selection of a new validation set was necessitated by the prior utilization of the CamCAN dataset, our original external validation set, in the training of the pyment model^23^. In this comparison, our hybrid CNN-MLP algorithm achieved an MAE of 5.111 years, an RMSE of 6.531 years, and an R^2^ of 0.919, while the pyment algorithm outperformed the CNN-MLP with an MAE of 4.264 years, an RMSE of 5.664 years, and an R^2^ of 0.939. The superior performance of the pyment algorithm might be partly attributed to its considerably larger training dataset (n = 53,542), compared to our dataset (n = 2703).

### Performance of the brain age prediction algorithms following bias correction

**Figure 3:**
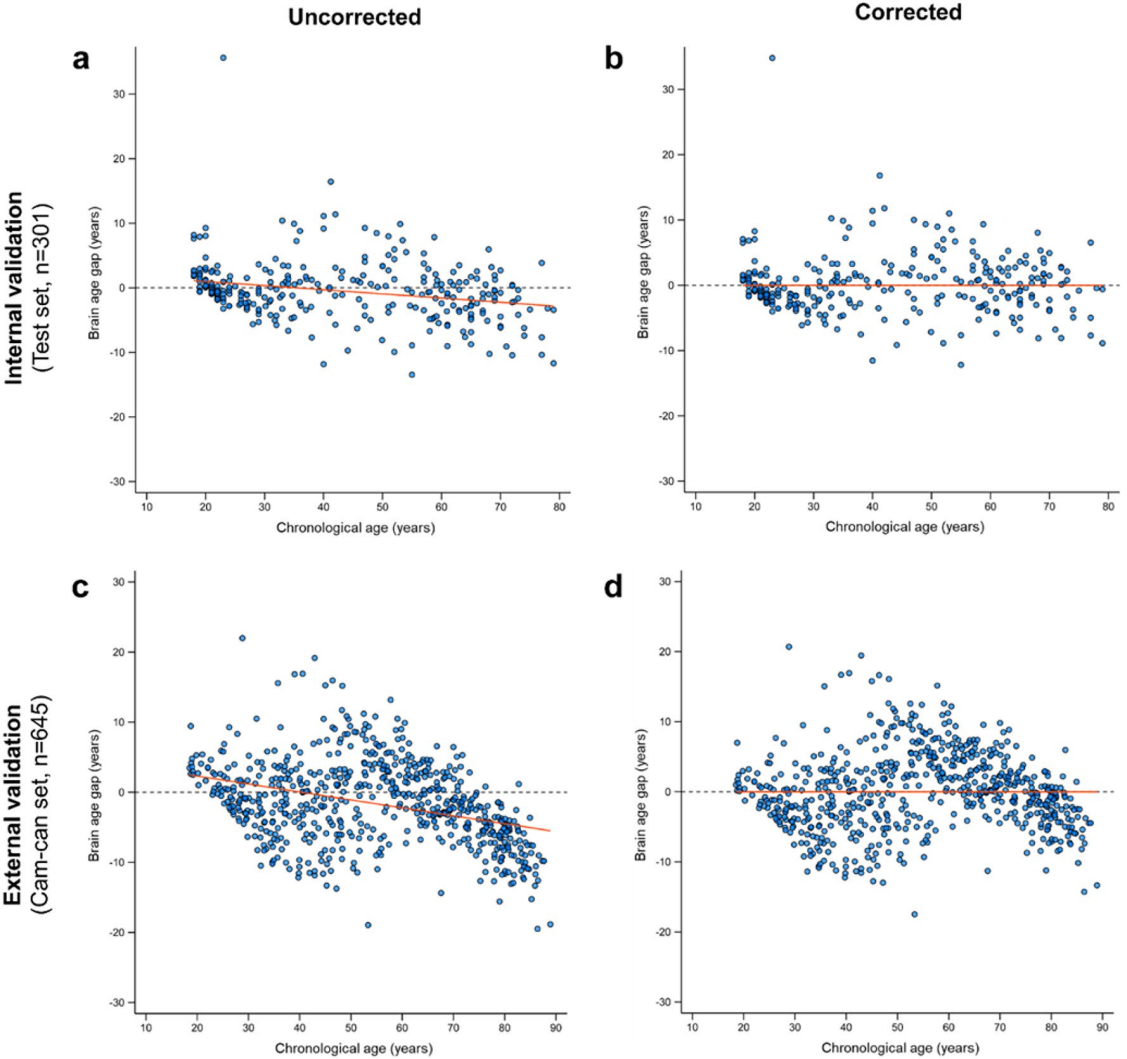
Scatter plots of brain age gap versus chronological age without and with linear bias correction. Results of brain age gap (predicted brain age—chronological age) without bias correction (**a**, **c**), and with bias correction (**b**, **d**). Scatter plots show the brain age gap, predicted by the proposed algorithm, versus chronological age in the test set (**a**, **b**), and in the CamCAN dataset (c, d). In all plots, the red lines indicate linear regression lines, and the dashed gray lines indicate ideal estimation references (y = 0). Abbreviations: CamCAN, Cambridge Centre for Ageing and Neuroscience.

The performance of the combined CNN-MLP algorithm, both before and after the application of bias correction, is detailed in Table 3 and Fig. 3. The application of linear bias correction improved the predictive performance of the combined CNN-MLP algorithm in both internal validation (n = 301) and external validation (n = 645).

**Table 3:** Predictive accuracy of the combined CNN-MLP algorithm without and with bias correction.

Input	Without bias correction			With bias correction		
	MAE (years)	RMSE (years)	R^2^	MAE (years)	RMSE (years)	R^2^
Internal validation (Test set, n = 301)	3.184	4.687	0.936	3.134	4.510	0.941
External validation (CamCAN set, n = 645)	4.910	6.148	0.891	4.313	5.546	0.911

*CamCAN* Cambridge Centre for Ageing and Neuroscience, *MAE* mean absolute error, *RMSE* root mean squared error, *R*^*2*^ coefficient of determination.

In the internal validation, the performance metrics included an MAE of 3.184 years, an RMSE of 4.687 years, and an R^2^ of 0.936 prior to bias correction (Fig. 3a) and an MAE of 3.134 years, an RMSE of 4.510 years, and an R^2^ of 0.941 after bias correction (Fig. 3b). In the external validation, the performance metrics were an MAE of 4.910 years, an RMSE of 6.148 years, and an R^2^ of 0.891 before bias correction (Fig. 3c) and an MAE of 4.313 years, an RMSE of 5.546 years, and an R^2^ of 0.911 after bias correction (Fig. 3d).

### Performance of the brain age prediction algorithms with data augmentation

To evaluate the impact of training set enhancement on brain age prediction accuracy, we conducted supplementary analyses using an augmented dataset. This dataset was randomly augmented with a 30% probability, resulting in the generation of additional synthetic images. The augmentation protocol encompassed 3D rotations within a − 10 to 10° range and translations between − 10 and 10 voxels.

The performance of the algorithms on the internal validation set yielded an MAE of 3.283 years, an RMSE of 4.726 years, and an R^2^ of 0.932. For external validation, the results showed an MAE of 4.945 years, an RMSE of 6.313 years, and an R^2^ of 0.885. These findings are detailed in Supplementary Table S3.

### Visualization of critical brain regions for age prediction

**Figure 4:**
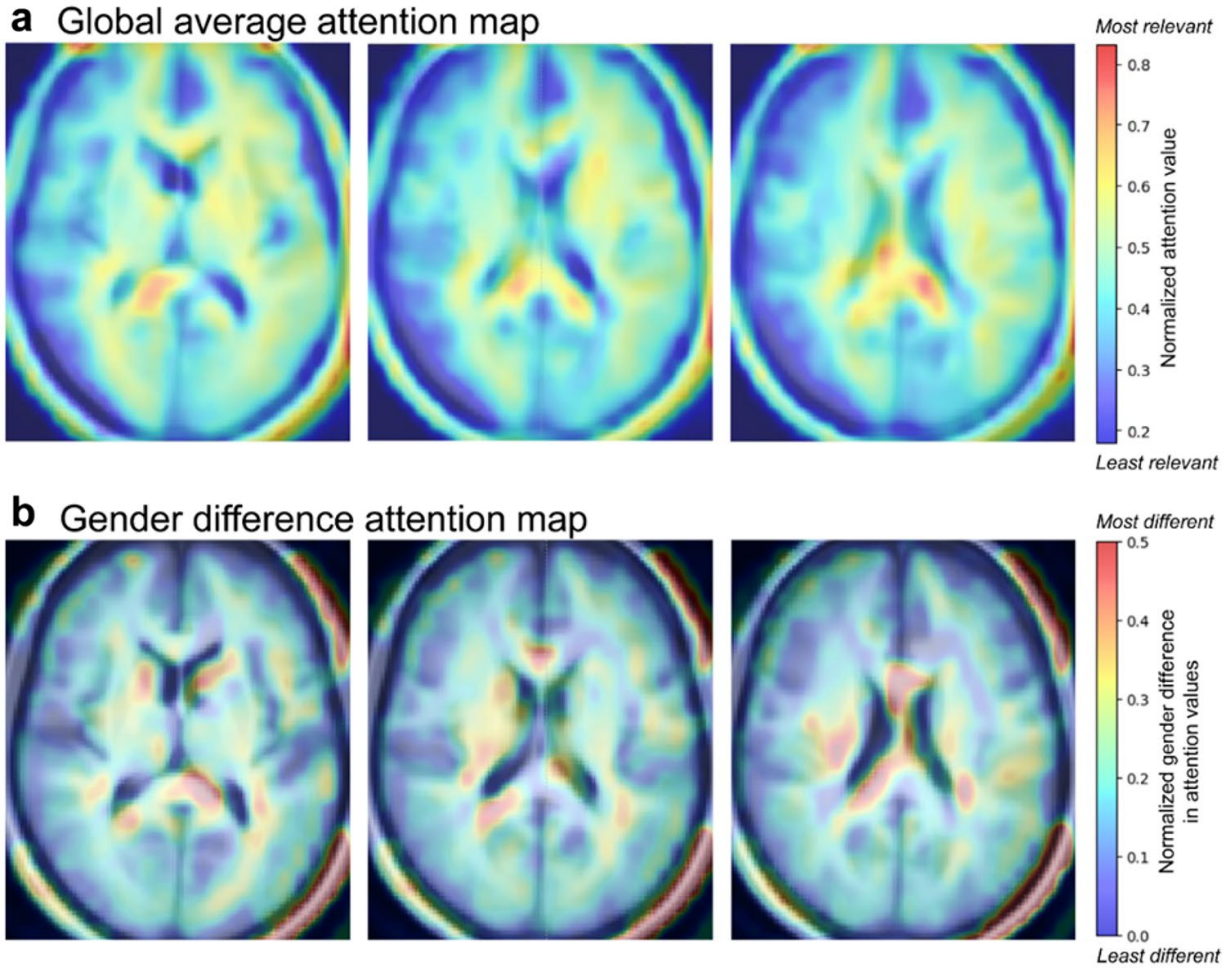
Visualization of critical brain regions for age prediction utilizing Grad CAM. (**a**) A global average attention map was created from the entire test set (n = 301). Regions marked with higher values, closer to red on the map, signify a greater contribution of those regions to age prediction. (**b**) A gender difference attention map was generated by subtracting the average attention map of female (n = 153) from that of male (n = 148). Regions marked with higher values, closer to red on the map, indicate stronger gender-specific influences of those regions on age prediction. All attention maps are overlaid over the averaged brain MR image from the test set.

A global average attention map, obtained from the entire test set (n = 301), revealed pronounced activation in the corpus callosum, internal capsule, and brain regions adjacent to the lateral ventricle (Fig. 4a). These findings suggest that these specific areas contribute more significantly to age prediction compared to other regions of the brain.

The gender difference attention map (Fig. 4b), generated by subtracting the average attention map of females (n = 153) from that of males (n = 148), demonstrated that the regions with the most distinct gender-specific contribution to age prediction were congruent with those highly activated in the global average attention map derived from the total sample.

### Application of brain age prediction algorithm to the MCI and AD groups

**Figure 5:**
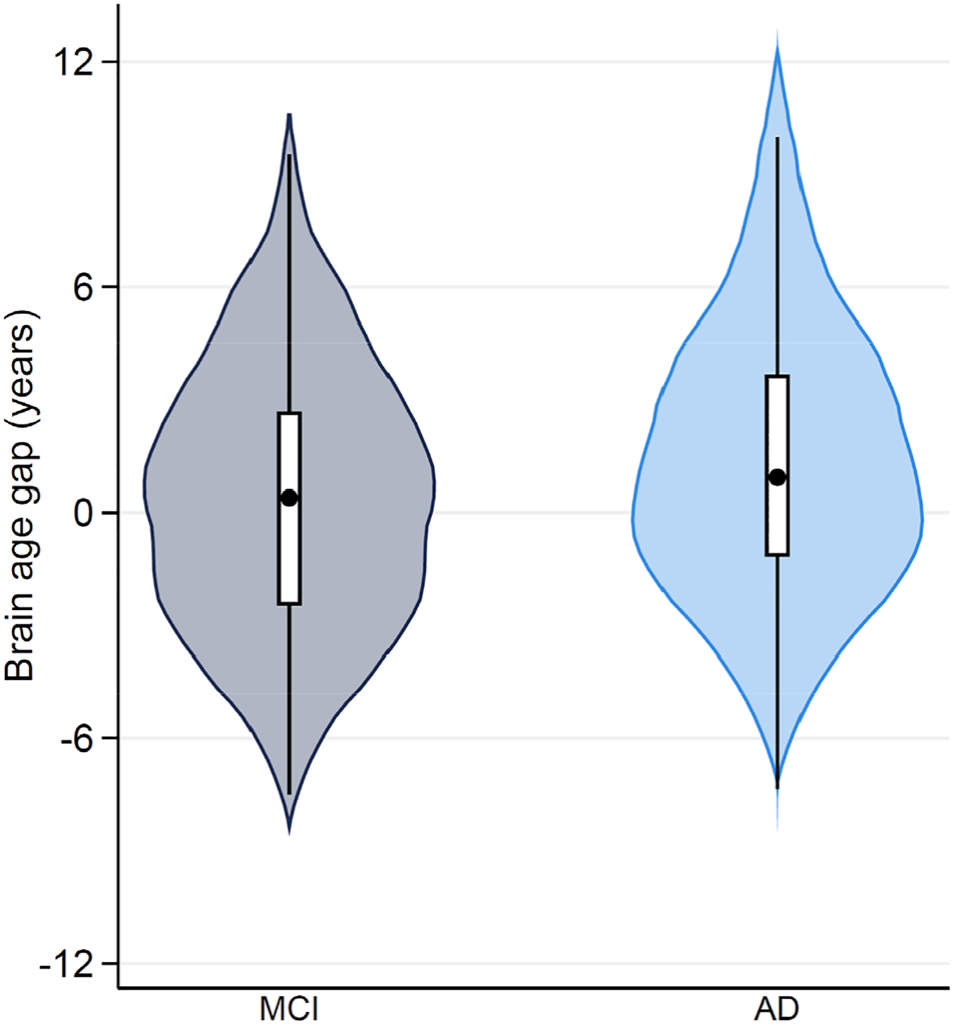
Distribution of brain age gap by clinical diagnosis. The mean (SD) brain age gaps were 0.413 (3.515) years and 1.393 (3.606) years for the MCI and AD groups, respectively. A significant difference in brain age gaps between the MCI and AD group was identified (*t* = − 2.673, *P* = 0.008). Abbreviations: AD, Alzheimer's disease; MCI, mild cognitive impairment; SD, standard deviation.

Employing our combined CNN-MLP algorithm, we estimated the brain age for patients with mild cognitive impairment (MCI, n = 208) and those diagnosed with Alzheimer's disease (AD, n = 172), as depicted in Table 4. The mean (SD) brain age gaps were calculated as 0.413 (3.515) years for the MCI group and 1.393 (3.606) years for the AD group, respectively (Fig. 5). A significant difference in brain age gaps between the MCI and AD groups (*t* = − 2.673, *P* = 0.008) was identified. This finding highlights the ability of our current brain age prediction model to efficiently differentiate between the two disease groups, underscoring its clinical relevance.

**Table 4:** Brain age estimation in patients with MCI and AD.

Group	n	Male	Age	Brain age	Brain age gap
		n (%)	Mean ± SD (range)	Mean ± SD (range)	Mean ± SD (range)
MCI	208	122 (58.7)	74.5 ± 7.3 (55.0–88.0)	74.8 ± 7.9 (52.7–90.8)	0.413 ± 3.515 (− 7.505–9.526)
AD	172	94 (54.7)	75.5 ± 7.5 (55.0–91.0)	76.9 ± 8.2 (49.7–95.7)	1.393 ± 3.606 (− 7.347–11.747)

Data were sourced from the ADNI1 dataset. For both the MCI and AD groups, brain age was determined using the combined CNN-MLP algorithm. The brain age gap was computed by determining the discrepancy between chronological age and the estimated brain age.

*AD* Alzheimer’s disease, *ADNI1* Alzheimer’s Disease Neuroimaging Initiative 1, *CNN* convolutional neural network, *MCI* mild cognitive impairment, *MLP* multi-layer perceptron, *SD* standard deviation.

## Discussion

By concatenating sex information with structural brain MRI data, the combined CNN-MLP algorithm exhibited higher accuracy in brain age prediction, in contrast to the CNN-only algorithm that relied solely on T1-weighted images. Furthermore, the combined CNN-MLP algorithm demonstrated superior predictive performance for brain age relative to the previously validated algorithms for brain age prediction, such as the brainageR model^15^.

In the present study, the hybrid architecture of the CNN-MLP algorithm was effective in achieving high accuracy for brain age prediction, a finding in line with recent research, evidencing enhanced performance and broader applicability through the synergistic use of multiple algorithms to proficiently manage diverse input types^17–19,26–28^. Specifically, the concatenating of the CNN algorithm with the MLP algorithm resulted in superior model performance, effectively accommodating factors that influence brain age, such as gender, site identification, and scanner information^9,29–31^. The hybrid CNN-MLP model, adept at merging x-ray images with numerical and categorical medical data, revealed a substantial 5–10% enhancement in discerning COVID-19 infection compared to existing models^17^. Our supplementary findings highlight the greater efficiency of the hybrid CNN-MLP algorithm over the CNN model augmented with an additional linear fully-connected layer, especially in processing sex information. The MLP algorithm's relatively streamlined structure, in comparison to other deep learning algorithms, may yield benefits such as reduced computational time and load in the creation of combined models^22,28^. Consequently, in terms of clinical flexibility and scalability, the pairing of CNN and MLP algorithms might offer a strategic advantage in handling complex data, including datasets containing images and varied clinical details^17–19,26–28^.

It is noteworthy that the proposed hybrid deep learning model takes into account both sex information and brain structural images when constructing the model. This is in contrast to other brain age prediction models that have subsequently corrected for sex during the validation process^14^. Given that sex has been shown to affect regional brain volumes^11,12,32,33^ and neurodegenerative changes^34,35^, in distinct and influential ways, integrating brain structures and sex information may bolster the model's efficacy in predicting brain age. This idea is supported by the fact that the CNN-MLP algorithm demonstrated superior predictive performance compared to the CNN algorithm, which relied solely on the T1-weighted image^22^. Our model bears resemblances to the innovative 3D convolutional network, the two-stage-age network (TSAN), which integrates MR images and sex labels as input variables^13^. However, TSAN diverges from our approach by incorporating a two-stage cascade architecture, wherein the initial age estimate is refined by a secondary network, adding an additional layer of analysis. This refinement enables TSAN to achieve significant accuracy, as evidenced by an MAE of 2.428 using a dataset of 6,586 subjects. To potentially improve our model’s accuracy, we undertook a supplementary analysis by incorporating a two-stage prediction method similar to that of TSAN (Supplementary Fig. S2, Supplementary Table S2). This adaptation of our CNN-MLP algorithm, to include a two-stage prediction process, yielded an MAE of 2.253 years, demonstrating improved performance closely paralleling that of the TSAN model.

Additionally, our findings reveal that the utilization of minimally preprocessed T1-weighted images in the combined CNN-MLP algorithm yielded better results than those of the tissue-segmented T1 images utilized in the brainageR algorithm^15^. Given the clinical importance of saving time and simplifying neuroimaging preprocessing, the current brain age prediction model, which employs minimally preprocessed T1-weighted images, can be applied efficiently in clinical environments^36,37^.

Considering that sex information is complexly and variably reflected in regional brain structures^11,12,21,32,33^, pinpointing the exact brain structural patterns displaying sex effects in influencing model performance has not reached a consensus, and findings have been inconsistent^38,39^. Within this framework, the present algorithm is able to simultaneously reflect whole-brain structural features to identify the sex-related pattern of aging in the brain, using minimally preprocessed neuroimaging in conjunction with sex information.

The proposed hybrid deep learning model was corrected for linear bias, utilizing individual neuroimaging during the modeling process, which enhanced the predictive accuracy for brain age. Although bias correction is critical for achieving both high accuracy and stability in brain age prediction^14,40,41^, most statistical corrections have been conducted based on chronological ages following modeling^7,40–42^. In this study, linear bias correction improved the predictive performance, reducing variance; the predicted brain age was refined by subtracting the offset corresponding to the brain age gap^40^. It may be inferred that linear bias correction can counter underfitting due to regression dilution and the non-Gaussian age distribution of the proposed model. Specifically, an incrementally increased brain age gap at the youngest and oldest extremities, along with a higher prediction error for individuals older than 50 years of chronological age, have been noted due to inter-individual variations in biological aging and biases in linear regression (e.g., linear regression toward the mean, attenuation)^43,44^.

To identify the brain regions that significantly influence age prediction, we utilized the Grad-CAM, an explainable artificial intelligence method, to create a voxel-wise average attention map^45^. In line with previous studies^46–49^, we discerned that the corpus callosum, internal capsule, and areas near the lateral ventricle were significant contributors to age prediction. Given the established significance of ventricular enlargement and atrophic changes near the lateral ventricle in the brain aging process^50,51^, these regions likely play a vital role in enhancing model performance.

Moreover, our findings regarding gender differences in the attention maps corroborate previous research on gender-specific aging processes in white matter areas, particularly around the corpus callosum and internal capsule^11,32,34^. This underscores the value of incorporating sex information into the brain age model to augment its predictive accuracy.

It is important to note that our hybrid CNN-MLP algorithm accurately predicted brain age in healthy individuals and also adeptly differentiated between the two neurodegenerative disease groups, MCI and AD, by identifying variances in their brain age gaps. The extent of brain age gaps for MCI and AD, as determined by our hybrid CNN-MLP model, aligns with that previously documented by Karim et al.^52^. From a scientific research perspective, using the brain age prediction model to analyze disease groups, especially in computing brain age gaps, greatly enhances our understanding of the model’s clinical implications^7,53^. Consequently, the current data robustly support the clinical relevance of our hybrid CNN-MLP model, specifically in the field of neurodegenerative diseases.

The following limitations should be considered in interpreting the current results. It is important to understand changes in brain structure and function that are associated with the variations in sex hormones^12^. Numerous estrogen receptors are found within the central nervous system, hence differences are evident between childbearing-age women and menopausal women^54^. Specifically, it has been noted that the characteristics of the brain consistently change in tandem with the menstrual cycle^55^. However, since information such as menopausal status and menstrual cycle of female subjects were not obtained from the database utilized in this study, the related factors potentially impacting prediction performance were not completely accounted for. Therefore, future investigations that include sex hormonal information alongside neuroimaging may offer additional insights into the effects of gender on brain aging^54,55^.

While the performance of the model that employs the combined CNN-MLP algorithm did exceed that of the CNN-only algorithm, this improvement did not attain statistical significance. Our findings align with numerous previous studies on brain age prediction models, where numerical differences in model performance were noted but without reaching statistical significance, hinting at performance enhancement^15,40,42,53^. Nonetheless, future research is warranted to confirm the improved performance of the combined CNN-MLP model, incorporating high-resolution structural images and sex information, through more rigorous statistical evaluations^15,40,42,53,56,57^.

Moreover, recent algorithms that employed more than 10,000 brain images for training have accomplished brain age prediction with an impressive MAE of less than three years^23^. In line with this, the pyment model, which benefited from a significant training set (n = 53,542), surpassed our CNN-MLP algorithm, which was developed using a considerably smaller training set (n = 2703), in terms of predictive accuracy. Therefore, enriching the training set could potentially boost the performance of our proposed CNN-MLP algorithm in subsequent studies.

While the CamCAN dataset is recognized for its reflection of the general population in terms of demographic variables^58,59^, it should be noted that the generalizability of the model's performance across varied populations still demands further examination and validation in future investigations.

It is important to underline that our MLP algorithm solely utilized gender information for predicting brain age, not including several vital features such as scanner information or site identification. This limitation was in part due to the absence or ambiguity of relevant information in the available dataset. Considering the proven capability of the MLP algorithm in handling various types of biological information^60–62^, future work should include essential features such as gender, site identification, or scanner information, all known to influence brain age^32–35^. The integration of these features into the hybrid CNN-MLP algorithm may notably augment model performance.

It warrants emphasis that future research utilizing the hybrid CNN-MLP algorithm should carefully incorporate both genetic and environmental factors, due to their well-documented impacts on brain aging^63–66^. In alignment with this perspective, recent investigations have developed algorithms skilled in processing multimodal data. This approach provides a more comprehensive framework, integrating MRI data with other relevant variables. For example, Qiang et al.^64^ created an integrated CNN-MLP framework that effectively combined MRI data with clinical and APOE genetic markers, thereby enhancing the diagnostic accuracy for AD. This underscores the potential benefits of augmenting traditional imaging data with genetic and clinical information to enhance model performance. Similarly, Bintsi et al.^65^ demonstrated improved performance by concurrently integrating imaging and non-imaging variables, such as blood pressure, stroke history, and alcohol consumption, into brain age estimation models. These non-imaging environmental factors have previously been shown to have significant correlations with brain aging^65,66^. Employing a multimodal approach that considers both imaging and non-imaging genetic/environmental variables has been shown to improve the accuracy of brain age estimation^65^.

Furthermore, future research involving multimodal neuroimaging (for example, both functional and structural neuroimaging), feature selection, and optimal parameter tuning could refine and optimize the proposed CNN-MLP algorithm^67–69^.

In the current study, the hybrid CNN-MLP algorithm, utilizing the minimally preprocessed T1-weighted images along with sex information, showed higher accuracy in predicting brain age compared to the CNN-only algorithm. These findings may suggest that neuroanatomical changes in brain aging could intertwine with sexually dimorphic clinical features. Accordingly, the proposed CNN-MLP algorithm could broaden our understanding of individual brain aging patterns in the context of both normal and pathological aging and provide critical insights regarding sexually individualized interventions.

## Methods

### Data collection

The current study included 3004 T1-weighted images of healthy subjects, whose ages ranged from 18.0 to 86.3 years, sourced from various open neuroimaging databases (mean age = 42.1 years, standard deviation [SD] = 18.7; consisting of 1471 men and 1,533 women). We excluded individuals with significant neurological or psychiatric disorders. For the longitudinal databases that contained follow-up brain imaging, only the brain structural MRI images from the baseline assessment were utilized to prevent data leakage between the training and test sets.

The dataset was stratified according to each age bin to ensure an identical age distribution in both the training and test sets. It was randomly divided into the training set (n = 2703) and the test set (n = 301).

The databases included 1000 Functional Connectomes Project (1000 FCP, available at http://fcon_1000.projects.nitrc.org/fcpClassic/FcpTable.html)^70,71^, International Neuroimaging Data-Sharing Initiative (INDI, available at http://fcon_1000.projects.nitrc.org/indi/IndiPro.html)^71^, Information eXtraction from Images (IXI, available at https://brain-development.org/ixi-dataset), Open Access Series of Imaging Studies 3 (OASIS-3, available at https://oasis-brains.org/)^72^, OpenNeuro (available at https://openneuro.org/), and Cambridge Centre for Ageing Neuroscience (CamCAN, available at http://www.mrc-cbu.cam.ac.uk/datasets/camcan/)^73^.

The corresponding Institutional Review Boards of the aforementioned open databases (1000 FCP, INDI, IXI, OASIS-3, OpenNeuro, CamCAN) either provided waivers or granted approval for the submission of anonymized data. Written informed consent was obtained from each subject. This research was conducted in compliance with the Declaration of Helsinki. The databases and detailed information regarding the included subjects are provided in Table 5.

**Table 5:** Demographic information of the subjects from the five datasets.

Dataset	n	Age	Male	Age-male	Female	Age-female
		mean ± SD (range)	n (%)	mean ± SD (range)	n (%)	mean ± SD (range)
1000 FCP	939	28.6 ± 13.8 (18.0–85.0)	398 (42.4)	29.3 ± 13.8 (18.0–78.0)	541 (57.6)	28.1 ± 13.7 (18.0–85.0)
INDI	982	45.7 ± 16.6 (18.0–80.0)	444 (45.2)	44.6 ± 16.6 (18.0–80.0)	538 (54.8)	46.6 ± 16.5 (18.0–78.0)
IXI	461	53.6 ± 13.8 (20.0–86.3)	205 (44.5)	51.4 ± 14.4 (30.0–86.2)	256 (55.5)	55.3 ± 13.1 (20.0–86.3)
OASIS-3	218	65.7 ± 6.7 (42.0–79.0)	218 (100.0)	65.7 ± 6.7 (42.0–79.0)	0 (0.0)	–
OpenNeuro	404	38.0 ± 17.3 (18.0–84.0)	207 (51.2)	37.7 ± 16.8 (18.0–81.0)	196 (48.5)	38.3 ± 17.9 (18.0–84.0)
Total	3004	42.0 ± 18.6 (18.0–86.3)	1472 (49.0)	43.6 ± 18.7 (18.0–86.2)	1532 (51.0)	40.4 ± 18.3 (18.0–86.3)

*FCP* Functional Connectome Project, *INDI* International Neuroimaging Data-sharing Initiative, *IXI* Information eXtraction from Images, *n* number, *OASIS* Open Access Series of Imaging Studies, *SD* standard deviation.

### Data preprocessing

Data preprocessing was conducted using Statistical Parametric Mapping (SPM) 12 software (Wellcome Centre for Human Neuroimaging, London, UK). This process involved non-linearly registering T1-weighted images in native space to the Montreal Neurological Institute (MNI) standard space. Such normalization across various scanner types and acquisition protocols ensures consistent model training. The normalization process in SPM12 also incorporated corrections for MR gradient field deviations, employing "bias regularization" and "bias FWHM" options^74,75^. Subsequently, the processed images were resampled to a voxel resolution of 1.5 mm using cubic spline interpolation, yielding a field-of-view of 105 × 127 × 105.

### Brain age prediction algorithms

In this study, we employed a three-dimensional (3D) CNN architecture, utilizing minimally preprocessed T1-weighted images with a dimension of 105 × 127 × 105 for brain age estimation^15^. This architecture consists of sequential convolutional blocks, each encompassing a 3D convolution layer, batch normalization layer, rectified linear unit (ReLU) activation function, and a max pooling layer with a stride of two. The initial block incorporated eight feature channels, while subsequent blocks double this number to better capture the intricate nuances of brain structures ^15^.

Following the convolutional blocks, the output from the final block was flattened and directed into a dense layer with sixty-four neurons and ReLU activation. This was then succeeded by a batch normalization layer, a dropout layer with a rate of 0.3, and another dense layer with sixteen neurons, again activated by ReLU.

The MLP architecture, formulated to process categorical sex information, integrated a dense layer with sixteen neurons activated by ReLU, followed by another dense layer with four neurons, also under ReLU activation.

**Figure 6:**
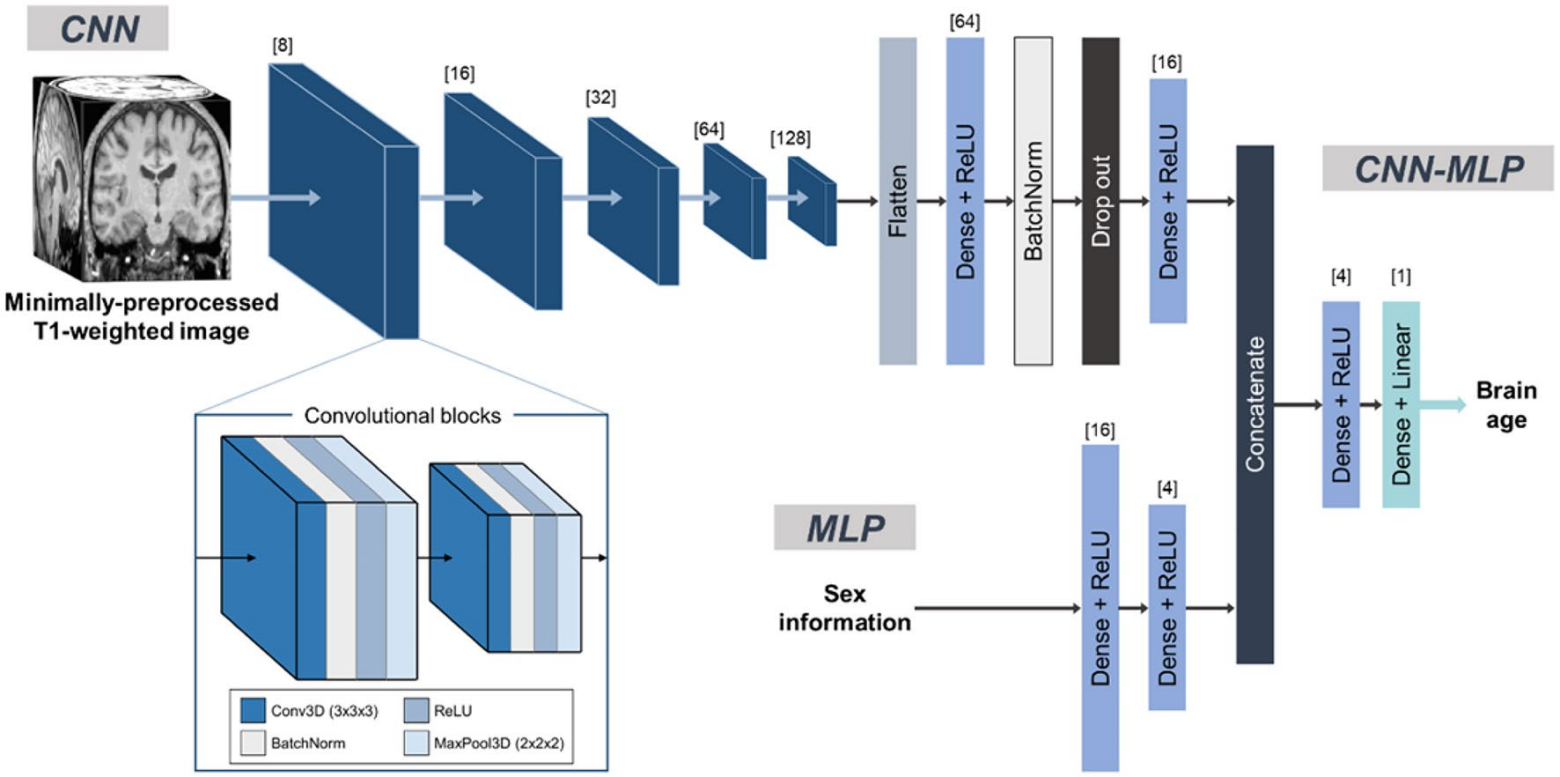
Overview of the proposed combined CNN-MLP algorithm for brain age prediction. The CNN architecture, designed for minimally preprocessed T1-weighted images, consists of repeated convolutional blocks, each with 3D convolutional layers, batch normalization, ReLU activations, and max pooling. After these blocks, the sequence includes a flattening layer, two dense layers interspersed with ReLU activations, batch normalization, and a dropout layer. The MLP, tailored for categorical sex information, features dense layers with ReLU activations. Both algorithms' outputs are merged by a concatenation layer, processed through two dense layers, with the final layer using a linear activation for brain age prediction. Abbreviations: 3D, 3-dimensional; BatchNorm, batch normalization; CNN, convolutional neural network; Conv, Convolution; MaxPool, max pooling; MLP, multi-layer perceptron; ReLU, rectified linear unit.

To create the combined CNN-MLP algorithm, the outputs from the concatenation layer were used as inputs. This concatenated input underwent processing through a dense layer with four neurons activated by ReLU, followed by an additional dense layer with a single neuron. Lastly, a linear activation function was applied to this final dense layer, deriving the predicted brain age. A schematic representation of the proposed architecture is depicted in Fig. 6.

The proposed algorithm was refined through hyperparameter tuning, a method renowned for boosting the accuracy of the brain age prediction model by adjusting key hyperparameters like batch size, epoch, learning rate, and neural network structural variables^76,77^. Hyperparameter tuning involves the utilization of different optimizers to stabilize the pattern of model updates^76^. Specifically, in this study, five optimizers were implemented across two combinations of learning rates and decay values: a learning rate of 0.01 with a decay of 0.003, and a learning rate of 0.001 with a decay of 0.0003. Among the investigated optimizers—adaptive gradient (Adagrad)^78^, adaptive moment estimation (Adam)^79^, Nesterov accelerated gradient (NAG)^80^, root mean square propagation (RMSprop)^80^, and stochastic gradient descent (SGD)^81^—the 10-fold cross-validation using Adam, with a learning rate of 0.001 and a decay of 0.0003, yielded the most favorable results. Comprehensive results for each of the five optimizers are detailed in Supplementary Table S1. It should be noted that, due to GPU constraints, the model was trained with a batch size of 16.

In addition, we constructed a CNN-only algorithm, trained exclusively with the minimally preprocessed T1-weighted images by using Adam with a learning rate of 0.001 and a decay of 0.0003, for the purpose of comparing its performance with the proposed combined CNN-MLP algorithm.

### Training and testing

To evaluate the performance of each algorithm, we utilized mean absolute error (MAE), root mean squared error (RMSE), and the coefficient of determination (R^2^) as performance metrics.

In this study, a 10-fold cross-validation scheme was applied to compare the performances of different methods: each algorithm was trained on nine randomly selected subsets, and then validated on the final subset, referred to as the validation set. The optimal algorithm was identified by evaluating the average performance metrics in 10-fold cross-validation.

Utilizing a computational framework comprising two NVIDIA Titan Xp GPUs with 12 GB memory, the training time for the CNN-MLP algorithm was approximately 6.94 h, whereas the CNN-only algorithm necessitated 5.28 h for training.

### External validation

The external validation of the proposed algorithms was performed using an independent dataset from the CamCAN (available at http://www.mrc-cbu.cam.ac.uk/datasets/camcan/)^73^. Recognized for its approximate reflection of the broader UK demographic profile, this dataset is deemed less biased and more generalizable^58,59^. Due to these attributes, the CamCAN set has been the preferred choice for external validation in numerous previous studies regarding brain age prediction models^58,59,82–84^. Specifically, the dataset, consisting of 645 individuals, demonstrated a balanced distribution of age (mean age = 54.7 years, SD = 18.6 years, range = 18.5–88.9 years, Supplementary Fig. S1) and gender (319 men, 49.5%, mean age = 55.1 years, SD = 18.4 years; 326 women, 50.5%, mean age = 54.3 years, SD = 18.8 years), enhancing its suitability for this study. We further validated our proposed model, the combined CNN-MLP algorithm, by contrasting its performance with well-established brain age prediction algorithm packages, specifically brainageR^15^. We selected brainageR for performance comparison because of the comparable size of its training dataset (brainageR, n = 3377 vs. our study, n = 2703) and its proven high, well-validated performance, making it a suitable benchmark^85^. According to Cole et al.^15^, the brainageR model was constructed using a computational setup that incorporated four NVIDIA Titan X GPUs. While their study^15^ did not specify the exact training duration, the application of Gaussian process regression (GPR) is known to reduce computational time compared to certain other deep learning algorithms with a similar level of performance.

In addition, we compared our model performance with another model, the pyment model^23^. The training process of the pyment model spanned approximately 70 h when using two NVIDIA V100 GPUs with 32 GB memory^23^. We selected it primarily for comparison because of its utilization of the CNN algorithm, a feature aligned with our current study. However, it is important to note that the pyment model was developed using a significantly larger, multisite dataset (n = 53,542), and thus surpassed various brain aging models, including ours, with an MAE of 2.47^23,30,86^. For this comparison, we employed a new, independent dataset comprising 200 healthy individuals (mean age = 57.6 years, SD = 23.0 years, range = 18.0–90.0 years; consisting of 93 men and 107 women) sourced from the ADNI1 and OASIS-1 databases, as the CamCAN dataset had been previously used in training the pyment model.

### Bias correction

The phenomenon of underfitting is frequently observed in brain age prediction models and can be attributed to factors such as regression dilution and non-Gaussian age distribution. Therefore, in the current study, a linear bias correction method^40^ predicted on the chronological age was employed to diminish the variance and enhance the prediction performance. The procedure entailed the following steps: Initially, the relationship between the offset, derived from the brain age gap (defined as the difference between the predicted brain age and the corresponding chronological age), and chronological age was established. Subsequently, the predicted brain age was refined by subtracting the identified offset.

### Visualization of critical brain regions for age prediction

To explore the specific brain regions that notably contribute to brain age prediction, we incorporated the explainable AI technique, gradient-weighted class activation mapping (Grad-CAM), into the CNN algorithm^45,87^. This approach facilitates the visualization of essential brain regions that are integral to the model's performance, employing a heat map^44^. Although Grad-GAM was originally devised for classification tasks^45^, we adapted it for regression algorithms, consistent with previous literature^88^,

In this particular application, we utilized Grad-GAM within a three-dimensional space to generate attention maps for individual brain images of the test set (n = 301), all registered to the MNI standard template. The values within attention maps were normalized within a range of 0–1, with a higher value denoting a more considerable contribution of a specific region to the overall brain age prediction. A global average voxel-wise attention map was subsequently created by averaging the individual attention maps.

To examine the influence of gender information on predictive performance, we crafted global average voxel-wise attention maps for both male (n = 148) and female (n = 153) samples in the test set. These attention maps could reveal gender-specific vital brain regions for age prediction. In our study, we visualized the gender-specific contributions of brain regions to age prediction by computing the differences between male and female attention maps. These difference values were normalized within a range of 0–1 and illustrated as a voxel-wise map. A higher value suggests a more marked gender difference in the contribution of brain regions to age prediction, possibly reflecting an augmented influence of gender information on age prediction.

### Brain age estimation in patients with MCI and AD

To investigate the clinical applicability of our brain age prediction model further, we employed the combined CNN-MLP algorithm to estimate the brain age in patients diagnosed with MCI (n = 208, MCI group) and AD (n = 172, AD group). The data utilized for these analyses were sourced from the ADNI1 database (available at https://adni.loni.usc.edu/about/adni1/)^89^. We determined the brain age gap, defined as the difference between chronological age and estimated brain age, for both the MCI and AD groups. Subsequently, we compared these brain age gaps between the two groups using an independent t-test.

### Supplementary Information

Supplementary Information.

## Data Availability

The datasets analyzed during the study are available in the following sources: 1000 Functional Connectomes Project (1000 FCP, available at http://fcon_1000.projects.nitrc.org/fcpClassic/FcpTable.html); International Neuroimaging Data-sharing Initiative (INDI) Prospective Data Sharing Samples (available at http://fcon_1000.projects.nitrc.org/indi/IndiPro.html); Information eXtraction from Images (IXI, available at https://brain-development.org/ixi-dataset/); Open Access Series of Imaging Studies (OASIS, available at http://www.oasis-brains.org/); Cambridge Centre for Aging and Neuroscience (CamCAN, available at https://www.cam-can.org/); Alzheimer’s Disease Neuroimaging Initiative 1 (ADNI 1, available at https://adni.loni.usc.edu/).
