# Supplementary material for: Brain age prediction using combined deep convolutional neural network and multi-layer perceptron algorithms
Source: Sci Rep. 2023 Dec 16;13:22388. doi: 10.1038/s41598-023-49514-2 (PMC10725434; doi:10.1038/s41598-023-49514-2)
Supplement: Supplementary file 1 — Supplementary Information. [file 41598_2023_49514_MOESM1_ESM.docx]

**Supplementary Information**

**Brain age prediction using combined deep convolutional neural network and multi-layer perceptron algorithms**

Yoonji Joo, Ph.D.^1†^, Eun Namgung, Ph.D.^2†^, Hyeonseok Jeong, Ph.D.^3^, Ilhyang Kang, Ph.D.^1^, Jinsol Kim, Ph.D.^1^, Sohyun Oh, M.S.^1,4^, In Kyoon Lyoo, M.D., Ph.D.^1,4,5^, Sujung Yoon, M.D., Ph.D.^1,4*^, Jaeuk Hwang, M.D., Ph.D.^6*^

^1^ Ewha Brain Institute, Ewha W. University, Seoul, South Korea

^2^ Asan Institute for Life Sciences, Asan Medical Center, Seoul, South Korea

^3^ Department of Radiology, Incheon St. Mary’s Hospital, College of Medicine, The Catholic University of Korea, Seoul, South Korea

^4^ Department of Brain and Cognitive Sciences, Ewha W. University, Seoul, South Korea

^5^ Graduate School of Pharmaceutical Sciences, Ewha W. University, Seoul, South Korea

^6^ Department of Psychiatry, Soonchunhyang University College of Medicine, Seoul, South Korea

^†^ These authors contributed equally.

^*^ Corresponding authors

**Supplementary Fig. S1.** Age distribution within the CamCAN dataset


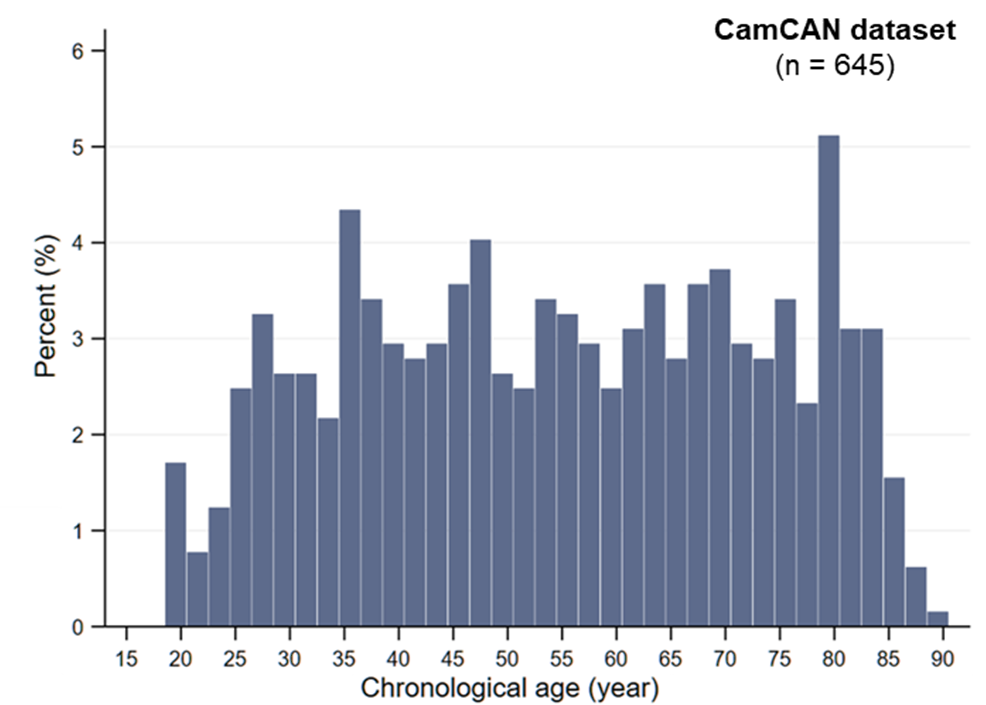


Abbreviations: CamCAN, Cambridge Centre for Ageing and Neuroscience; n, number.

**Supplementary Fig. S2.** Overview of the modified CNN-MLP algorithm with the integrated two-stage prediction process


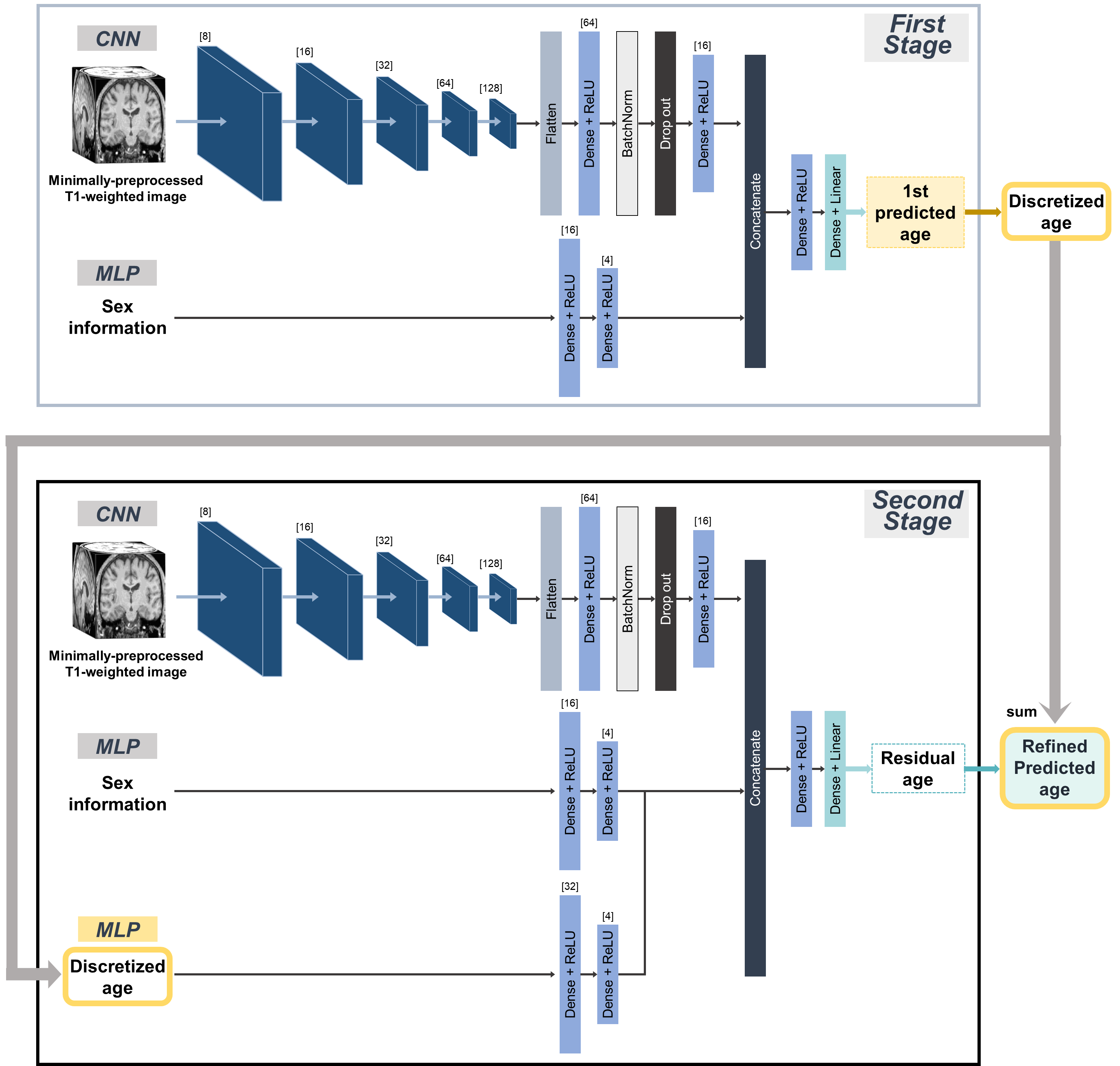


In this supplementary analysis, we incorporated a two-stage prediction process, akin to the two-stage-age network (TSAN) framework [1], into our CNN-MLP algorithm. Initially, the network utilized both brain imaging and sex data to predict brain age. This first-stage estimation was refined by the second-stage process, which employed the initial brain image and sex data together with the discretized brain age output from the first stage, to yield a more precise age determination.

Abbreviations: 3D, Three-dimensional; BatchNorm, batch normalization; CNN, convolutional neural network; Conv, Convolution; MaxPool, max pooling; MLP, multi-layer perceptron; ReLU, rectified linear unit.

**Supplementary Table S1.** Predictive accuracy in proposed models: evaluating five hyperparameter optimizers

| Optimizer | Learning rate | Decay | Performance metrics | | |
| --- | --- | --- | --- | --- | --- |
|  |  |  | MAE (years) | RMSE (years) | R^2^ |
| SGD | 0.01 | 0.003 | 3.631 ± 0.226 | 5.033 ± 0.440 | 0.927 ± 0.013 |
|  | 0.001 | 0.003 | 3.669 ± 0.281 | 5.035 ± 0.452 | 0.926 ± 0.014 |
| NAG | 0.01 | 0.003 | 3.567 ± 0.190 | 4.876 ± 0.256 | 0.931 ± 0.008 |
|  | 0.001 | 0.003 | 3.752 ± 0.286 | 5.098 ± 0.449 | 0.924 ± 0.014 |
| Adagrad | 0.01 | 0.003 | 4.099 ± 0.351 | 5.682 ± 0.923 | 0.905 ± 0.033 |
|  | 0.001 | 0.003 | 3.574 ± 0.139 | 4.949 ± 0.258 | 0.929 ± 0.009 |
| RMSprop | 0.01 | 0.003 | 3.507 ± 0.199 | 4.667 ± 0.472 | 0.933 ± 0.010 |
|  | 0.001 | 0.003 | 3.715 ± 0.252 | 5.072 ± 0.411 | 0.925 ± 0.012 |
| Adam | 0.01 | 0.003 | 3.529 ± 0.201 | 4.927 ± 0.495 | 0.930 ± 0.014 |
|  | 0.001 | 0.003 | 3.494 ± 0.228 | 4.689 ± 0.570 | 0.933 ± 0.012 |

Within the proposed CNN-MLP algorithm framework, model performance was enhanced through hyperparameter tuning employing five distinct optimizers. Performance metrics were ascertained via 10-fold cross validation applied to the training dataset.

Abbreviations: Adagrad, adaptive gradient; Adam, adaptive moment estimation; MAE, mean absolute error; NAG, Nesterov accelerated gradient; R^2^, coefficient of determination; RMSE, root mean square error; RMSprop, root mean square propagation; SGD, stochastic gradient descent.

**Supplementary Table S2.** Results of the modified CNN-MLP algorithm utilizing the two-stage prediction process

| Stage | Input | Performance metrics | | |
| --- | --- | --- | --- | --- |
|  |  | MAE (years) | RMSE (years) | R^2^ |
| First stage | T1-weighted image  + sex information | 4.910 | 6.148 | 0.891 |
| Second stage | T1-weighted image  + sex information  + discretized brain age by the first stage | 2.253 | 2.834 | 0.977 |

The performance of each stage was evaluated using the external validation dataset from the CamCAN set (n = 645).

Abbreviations: CamCAN, Cambridge Centre for Ageing and Neuroscience; MAE, mean absolute error; RMSE, root mean squared error; R^2^, coefficient of determination.

**Supplementary Table S3.** Predictive accuracy of the combined CNN-MLP algorithm with data augmentation

| Input | MAE (years) | RMSE (years) | R^2^ |
| --- | --- | --- | --- |
| Internal validation  (Test set, n = 301) | 3.283 | 4.726 | 0.932 |
| External validation  (CamCAN set, n = 645) | 4.945 | 6.313 | 0.885 |

Data augmentation, conducted with a 30% probability, comprised 3D rotations of −10° and 10°, along with translations from −10 to 10 voxels.

Abbreviations: CamCAN, Cambridge Centre for Ageing and Neuroscience; MAE, mean absolute error; RMSE, root mean squared error; R^2^, coefficient of determination.

**REFERENCE**

[1] Cheng, J. *et al*. Brain age estimation from MRI using cascade networks with ranking loss. *IEEE Trans. Med.* **40**, 3400-3412 (2021).
